# Supplementary material for: Generalised analytical method unravels framework-dependent kinetics of adsorption-induced structural transition in flexible metal–organic frameworks
Source: Nat Commun. 2023 Nov 8;14:6862. doi: 10.1038/s41467-023-42448-3 (PMC10632496; doi:10.1038/s41467-023-42448-3)
Supplement: Supplementary file 1 — Supplementary Information [file 41467_2023_42448_MOESM1_ESM.pdf]

Supplementary Information

Generalised analytical method unravels framework-dependent kinetics of adsorption-induced structural transition in flexible metal–organic frameworks

Sakanaka *et al.*

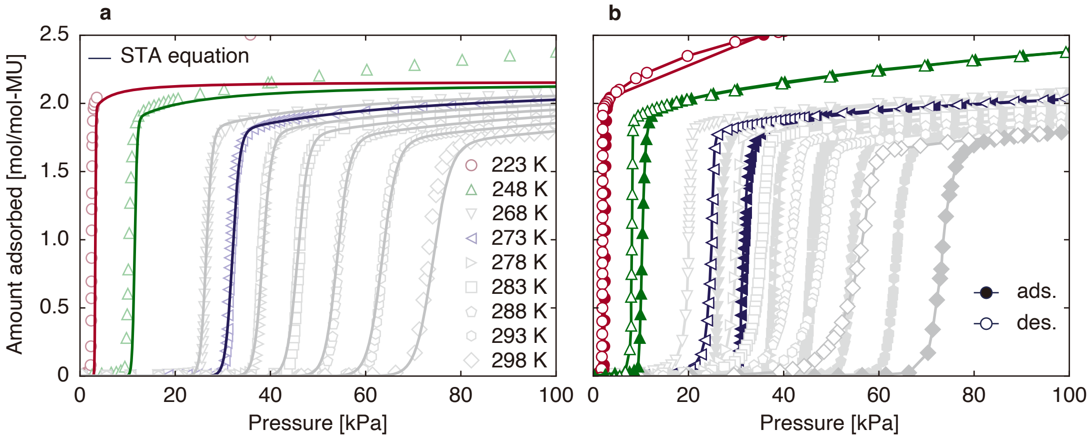

Supplementary Fig. 1 | Adsorption isotherms of CO_2 on ELM-11 at 223–298 K. a, Symbols and solid lines are experimental and calculated values by the STA equation, respectively, for adsorption branches. **b,** Adsorption/desorption isotherms at each temperature.

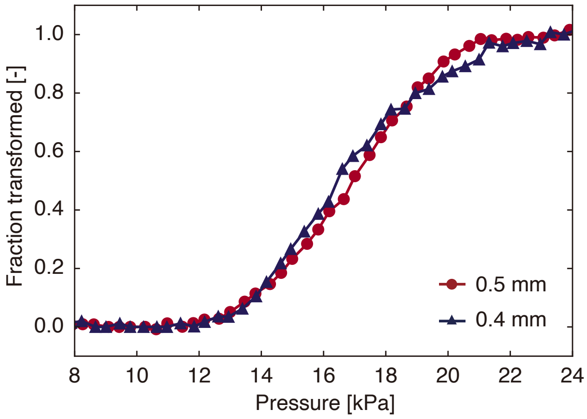

Supplementary Fig. 2 | Influence of inner diameter of glass capillary (0.4 and 0.5 mm) on time-resolved *in situ* XRPD measurements for ELM-11 ⊃ CO_2 at 248 K and 0.8 kPa s^{-1}.

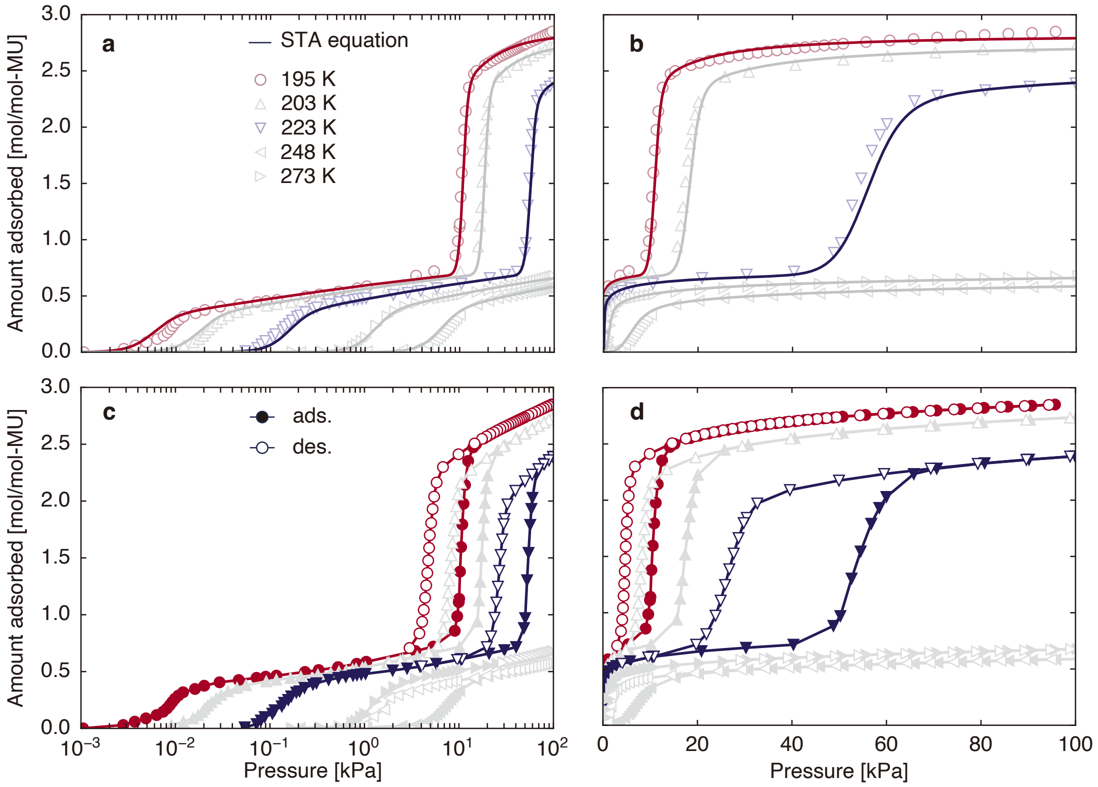

Supplementary Fig. 3 | Adsorption isotherms of CO_2 on MIL-53(Al) at 195–273 K. a and **b** are log scale and linear scale displays, respectively. Symbols and solid lines are experimental and calculated values by the STA equation, respectively. Note that the second NP–LP transition at 248 and 273 K occurs above 100 kPa. **c** and **d** are Adsorption/desorption isotherms at each temperature.

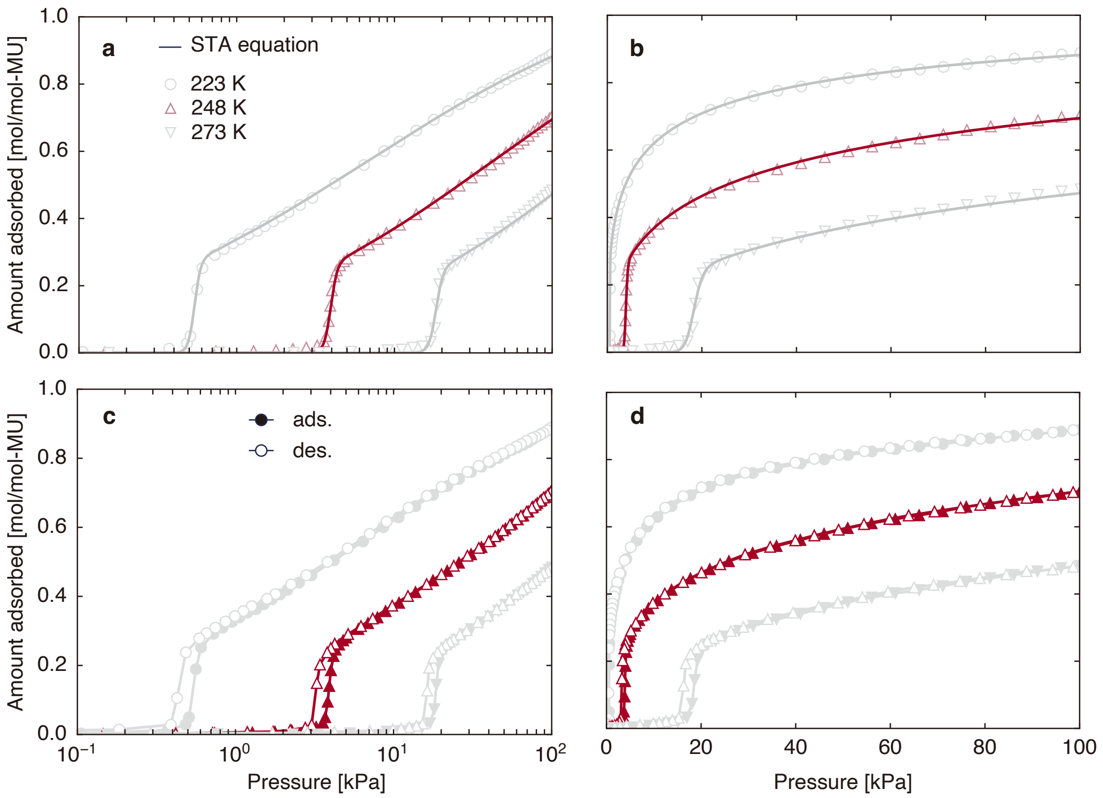

Supplementary Fig. 4 | Adsorption isotherms of CO_2 on CuFB at 223, 248, and 273 K. a and **b** are log scale and linear scale displays, respectively. Symbols and solid lines are experimental and calculated values by the STA equation, respectively. **c** and **d** are Adsorption/desorption isotherms at each temperature.

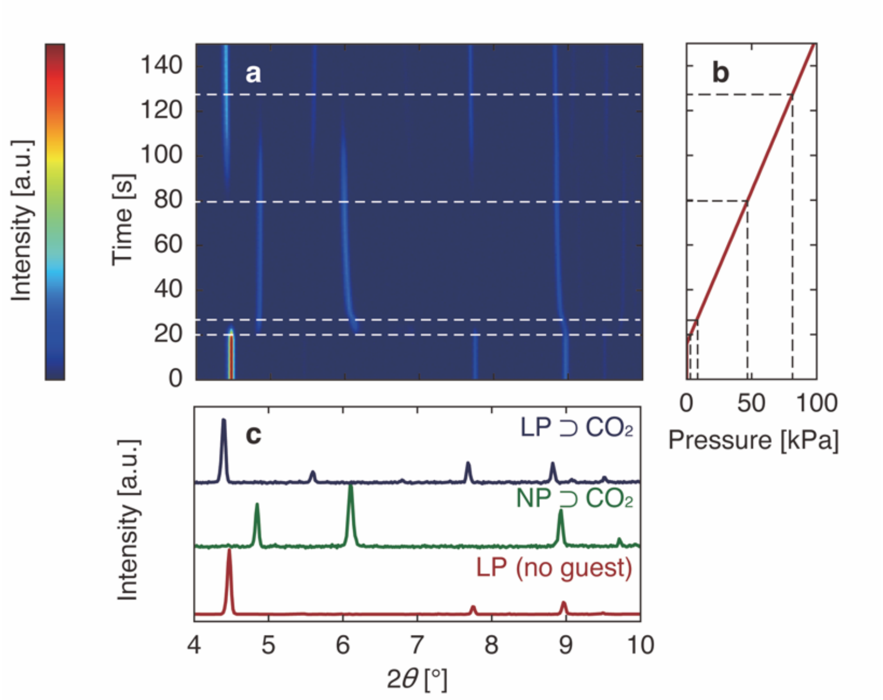

Supplementary Fig. 5 | Time-resolved in situ XRPD measurement. a, Color map of the XRPD pattern on MIL-53(Al) at 0.73 kPa s^{-1} and 223 K. **b,** The time dependence of the CO_2 pressure. **c,** XRPD patterns for LP phase (0 kPa), NP ⊃ CO_2 (10 kPa), and LP ⊃ CO_2 (100 kPa).

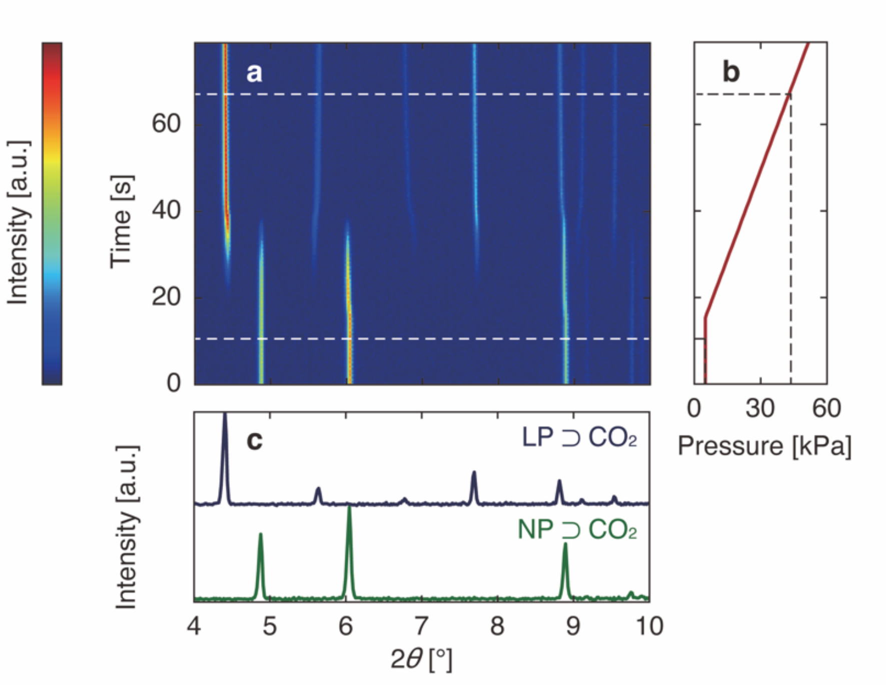

Supplementary Figure 6 | Time-resolved in situ XRPD measurement. a, Color map of the XRPD pattern on MIL-53(Al) at 0.73 kPa s^{-1} and 195 K. **b,** The time dependence of the CO_2 pressure. **c** XRPD patterns for NP ⊃ CO_2 (5 kPa) and LP ⊃ CO_2 (50 kPa).

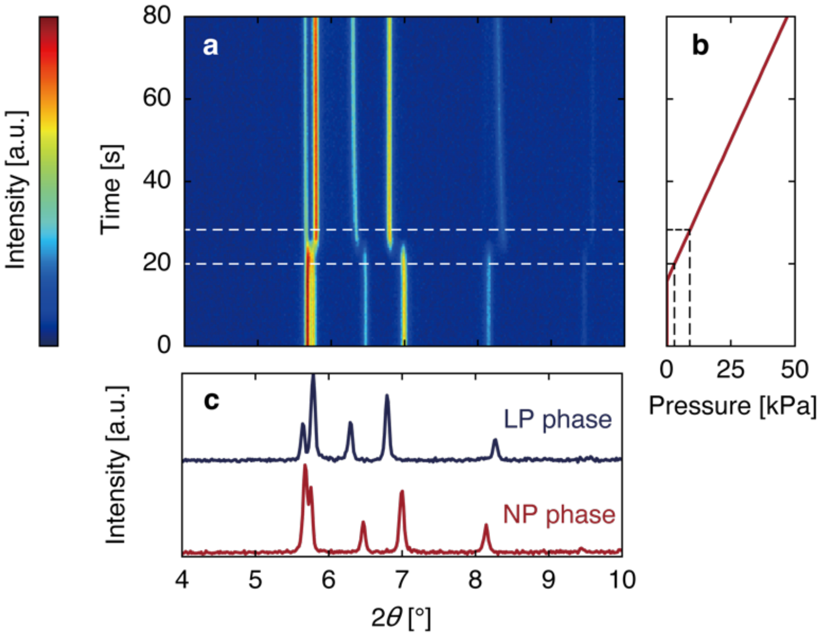

Supplementary Fig. 7 | Time-resolved in situ XRPD measurement for CuFB. a, Colormap of the XRPD pattern on CuFB at 0.73 kPa s^{-1} and 248 K. **b,** The time dependence of the CO_2 pressure. **c,** XRPD patterns for the open phase (100 kPa) and the closed phase (0 kPa).

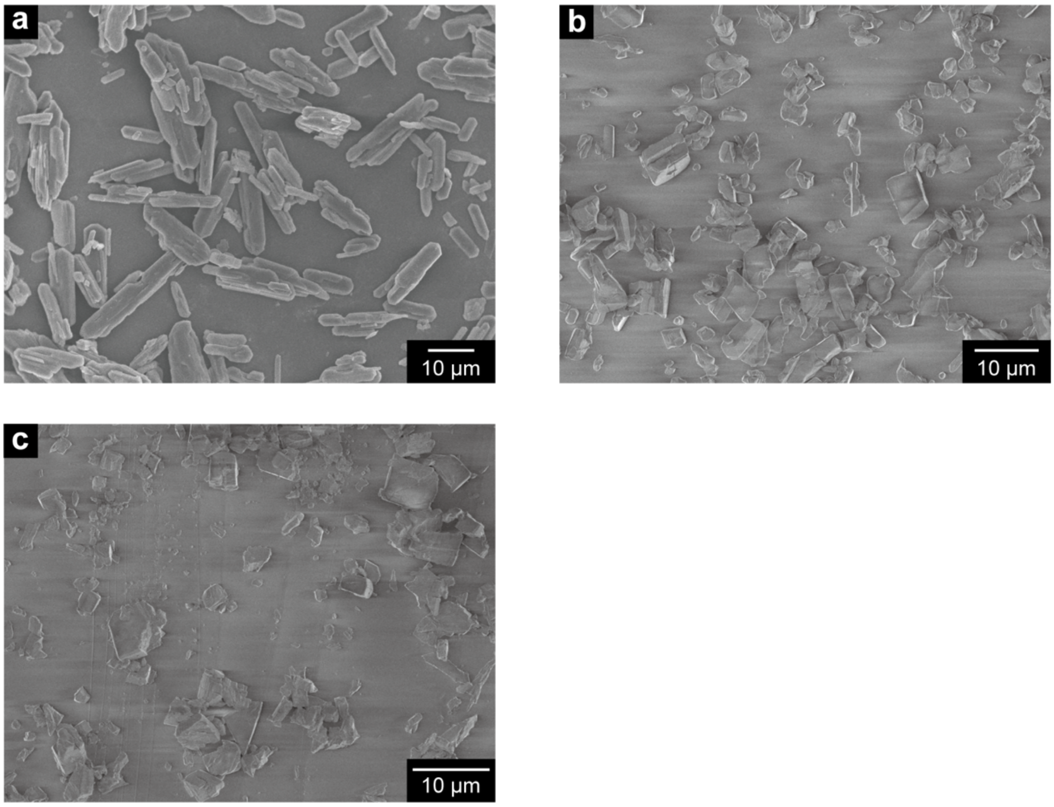

Supplementary Fig. 8 | Scanning electron microscopy images. a, pre-ELM-11. **b,** MIL-53(Al) **c,** CuFB.

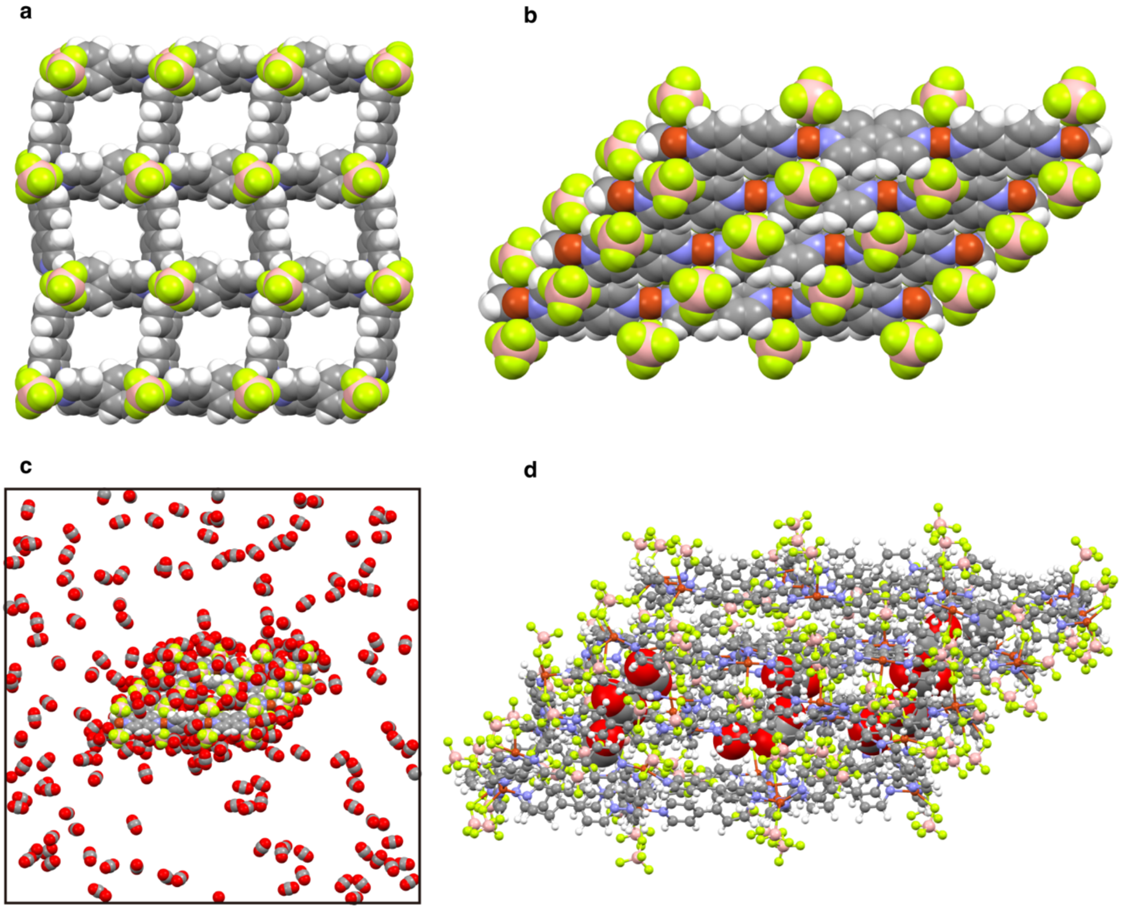

Supplementary Fig. 9 | Snapshots of molecular simulations. a, A layer structure of the ELM-11 cluster model. **b,** The ELM-11 cluster model. **c,** The initial structure of the MD simulation. **d,** The framework structure with encapsulated CO_2 molecules after the MD run.

Supplementary Table 1 | Parameters of the gate-type isotherm equation for CO_2 adsorption on ELM-11, CuFB, and MIL-53(Al).

	ELM-11	CuFB	MIL-53(Al)	unit
$n_{LP,ref}$	2.16	1.14	2.55	mol/mol-MU
$K_{LP,ref}$	5.96×10^{-4}	2.41×10^{-5}	5.6×10^{-5}	Pa^{-1}
Q_{LP}	30.5	33.7	22.5	kJ/mol
χ_{LP}	0.0	0.0	0.8	
$s_{LP,ref}$	1.0	1.95	0.63	
w_{LP}	0.0	0.49	2.0	
$n_{NP,ref}$	-	-	0.85	mol/mol-MU
$K_{NP,ref}$	-	-	2.0×10^{-3}	Pa^{-1}
Q_{NP}	-	-	32.3	kJ/mol
χ_{NP}	-	-	0.03	
$s_{NP,ref}$	-	-	3.2	
w_{NP}	-	-	0.38	
ΔU^{host}	13.6	1.92	–1.7	kJ/mol-MU
ΔS^{host}	17.9	2.9	4.18	$J\cdot mol\text{-}MU^{-1}\cdot K^{-1}$
β	24	80	10.4	
T_{ref}	248	248	223	K

Supplementary Note 1 | Reproducibility of TRXRD measurement.

To verify the reproducibility of TRXRD measurement, data were repetitively collected over 30 cycles under the same conditions (0.8 kPa s^{-1} and 248 K). ELM-11 was transformed into the closed state by room temperature (~293 K) evacuation before each measurement (Supplementary Fig. 9a). Although the shape of the profile did not change, the transition began at an earlier time with each repetition. Moreover, the (002) peak of the closed state shifted to a lower angle and decreased in intensity (Supplementary Fig. 9b). Thus, the atomic order of the closed structure was slightly disrupted with repeated measurements. Because a disordered closed phase is less stable than an ordered closed phase, the repeated measurements facilitated a transition to the relatively stable open phase, thereby reducing P_{gate} and shifting the transition time. To observe the kinetic process from a well-ordered closed state, the sample was annealed at 373 K before each measurement, which promoted closed-phase rearrangement. Consequently, the (002) peak regained its pre-measurement position and intensity, and the time evolution of α could be measured with good reproducibility (Supplementary Fig. 10b,c). The data discussed in this work were all obtained using the same treatment.

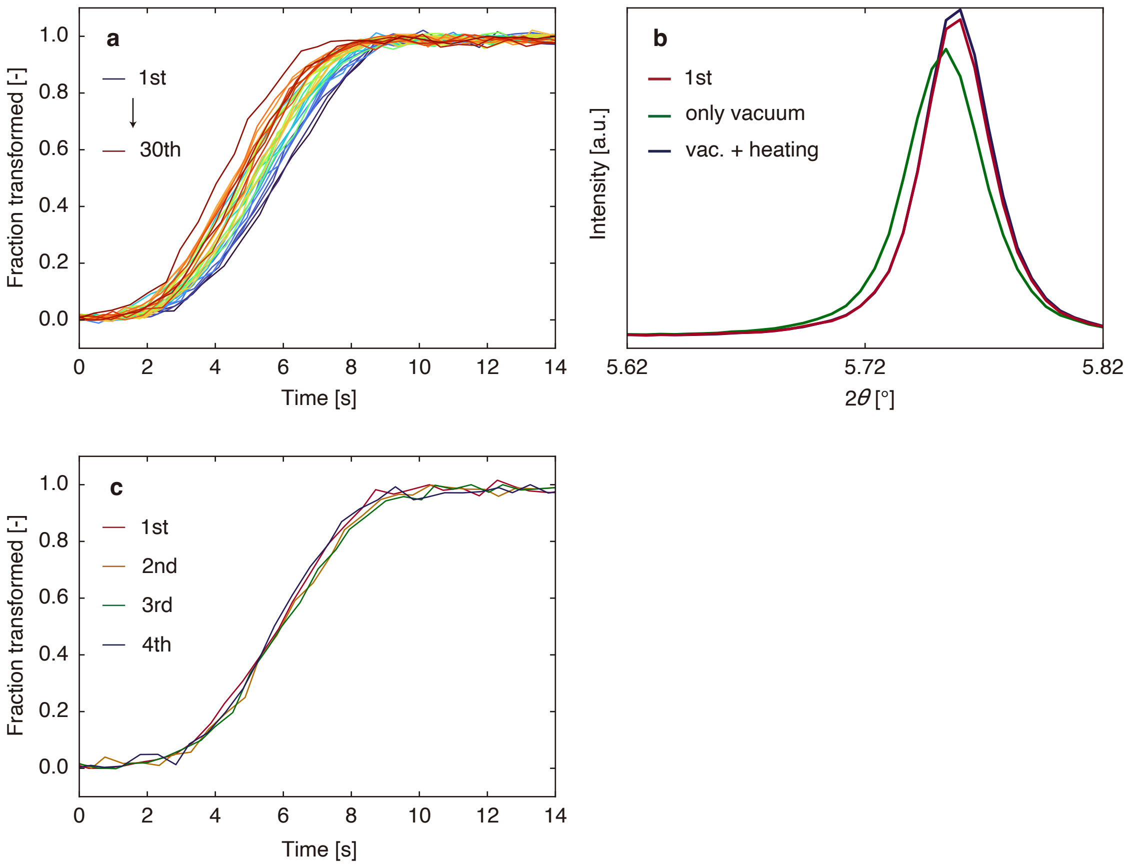

Supplementary Fig. 10 | Repetitive measurement results to confirm reproducibility. **a,** Time evolution of the fraction transformed at 0.8 kPa s^{-1} cycling the measurements under the same condition. **b,** XPRD patterns under the conditions of the first measurement (red), only evacuation (green), and annealing treatment with evacuation (blue). **c,** Reproducibility of the measurements when annealing treatment.

Supplementary Note 2 | Temperature dependence of the structural transition rate. Typically, the rates of physical phenomena, such as gas migration and reactions, increase with the temperature of the system. However, a previous study indicated an anti-Arrhenius relationship between the temperature and rate of CO_2 adsorption on ELM-11[1]. The variation of the gate opening rate of ELM-11 with temperature was investigated by recording the CO_2 adsorption rate at 223, 248, and 273 K (Supplementary Fig. 11a–c, which is the same as Fig. 4 in the manuscript). Although sigmoidal profiles were observed at all temperatures examined, the slope decreased with increasing temperature, indicating a longer duration of transition possibly due to the smaller adsorption isotherm slopes at higher temperatures (Supplementary Fig. 1). The experimental data fitted well to Model **AC** (solid curves in Supplementary Fig. 11a–c) with the rate constants k_1 and k_2 listed in Supplementary Table 2. The obtained rate constants did not follow the typical Arrhenius-type relationship and appear to be approximately constant with respect to temperature. In fact, Supplementary Fig. 11d–f shows curves of Model **AC** using the rate constants determined at 248 K, resulting in a reasonably good fit to the experiments, although only the discrepancy between the data at 0.8 kPa s^{-1} and 273 K was relatively large. Therefore, the previously reported decrease in the rate of CO_2 adsorption on ELM-11 with increasing temperature[1] can be attributed to an increase in $P_{gate}(T)$ and consequent decrease in the pressure difference, $P - P_{gate}(T)$. Thus, the parameters of the kinetic model can be determined from a single temperature condition, which would be useful for engineering applications.

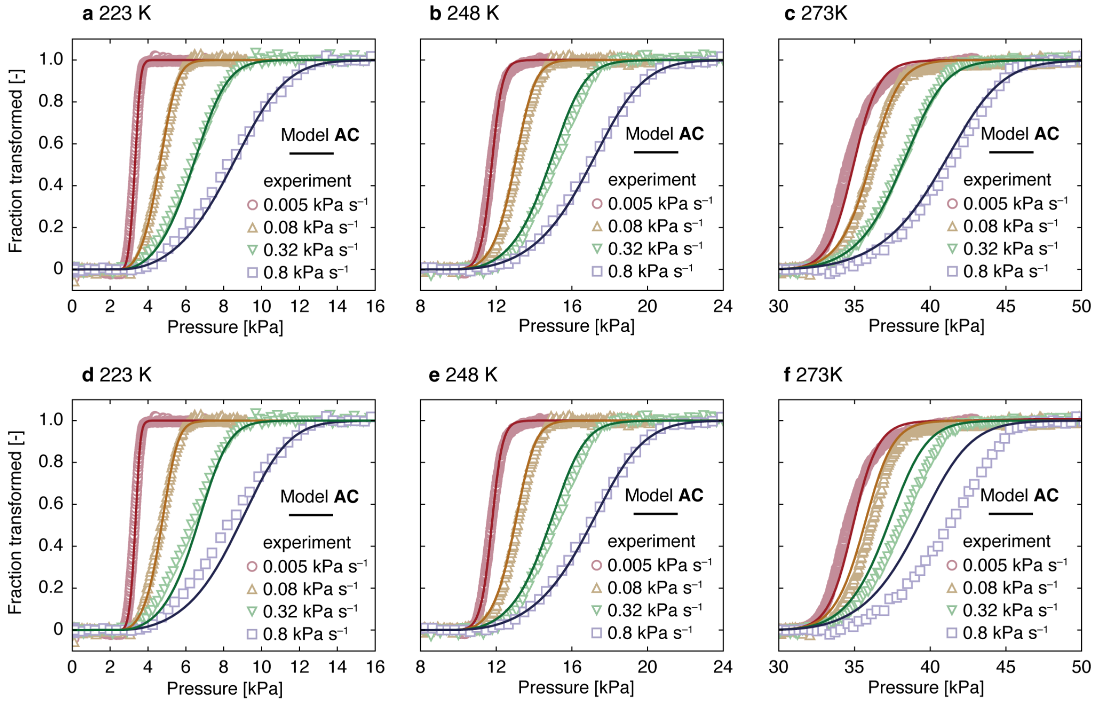

Supplementary Figure 11 | Derived structural transition rate model and its temperature dependence. a–c, Relationships between the fraction transformed and pressure obtained from TRXRD measurements (markers) and results calculated using the structural transition rate model (curves) at 223, 248, and 273 K, respectively. **d–f,** Those of **a–c** where Model **AC** is calculated using the rate constants determined in **b**.

Supplementary Table 2 | Estimated rate constants of Model AC for ELM-11⊃CO_2.

Temperature [K][a]	k_1 [kPa^{-1} s^{-1}]	k_2 [kPa^{-1} s^{-1}]
223 (222)	0.062	0.020
248 (248)	0.075	0.014
273 (275)	0.053	0.007

[a]The values in parentheses were used in fitting for $h(\alpha, T) = P_{\mathrm{gate}}(\alpha, T) = \sigma(P, T)^{-1}$.

Supplementary Note 3 | Kinetic analysis for MIL-53(Al) and CuFB.

The TRXRD data of ELM-11 indicates that the rate of structural transition is proportional to the differential pressure $P - P_{\mathrm{gate}}(\alpha)$. This fact allows us to estimate the function shape of $g(\alpha)$ (i.e., the reaction model) based on the TRXRD data at a single pressurization rate, using the following equation (derived from equations (3) and (4)):

$$g(\alpha) = \frac{v_{\mathrm{p}}}{P - P_{\mathrm{gate}}(\alpha)} \frac{\mathrm{d}\alpha}{\mathrm{d}P} \tag{S1}$$

For example, the red circle in Supplementary Fig. 12 shows the result of ELM-11 at 0.32 kPa s^{-1} (corresponding to the green marker in Fig. 4), indicating that g is a quadratic function of α (i.e., Model **AC**). This simplified analysis was applied to MIL-53(Al) and CuFB as shown in Supplementary Fig. 12, where the plots were obtained using the TRXRD data at 0.29 kPa s^{-1}. $g(\alpha)$ of the first LP→NP transition of MIL-53(Al) decreases linearly as α increases. Therefore, Model **FO** was selected ($g(\alpha) = k_{\mathrm{FO}}(1 - \alpha)$). Conversely, for the second NP→LP transition of MIL-53(Al) and transition of CuFB, the transition rate was found to be almost independent of α, leading to Model **ZO** ($g(\alpha) = k_{\mathrm{ZO}}$). After determining the shape of g(α) (the reaction model) based on this analysis, rate constants were optimized so that the theoretical α vs P plots for three pressurization rates could be fitted to those obtained from TRXRD measurements (Fig. 6f,i,l).

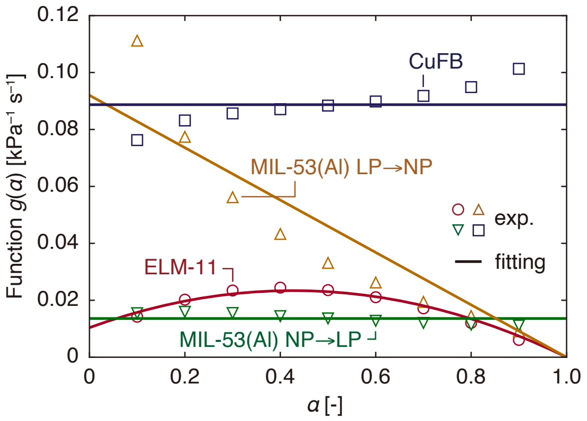

Supplementary Figure 12 | Relationship between the function $g(\alpha)$ obtained from equation (S1) and α. Each solid line was obtained by least-squares fitting of the corresponding model.

Supplementary Note 4 | Synthesis of CuFB.

Copper (II) nitrate hemi(pentahydrate) [$Cu(NO_3)_2 \cdot 2.5H_2O$, 98.0%], fumaric acid [H_2FMA, 98.0%], and 1,2-bis(4-pyridyl)ethylene [bpe, 98.0%] were purchased from Sigma-Aldrich Co. LLC., and FUJIFILM Wako Pure Chemical Corporation, and Tokyo Chemical Industry Co., Ltd., respectively. Methanol (99.8%) and acetone (99.5%) were purchased from Kishida Chemical Co., Ltd. Deionized water was prepared by using an arium mini plus (Sartorius) water purification system and was used for all experiments. CuFB particles were synthesized by using the processes reported by Chen *et al*[2]. H_2FMA and bpe solution were dissolved in 300 mL of water to a concentration of 14 mM and 7.1 mM, respectively. The solution were heated to approximately 360 K with stirring at 1350 rpm on a hotplate, and $Cu(NO_3)_2 \cdot 2.5H_2O$ (75mL, 57 mM) was added to the solution. The mixture was heated at 373 K in an oven for 24 h. The blue-green particles were collected by vacuum filtration and washed with deionized hot water (approximately 360 K) and methanol (approximately 333 K). To completely remove the guest molecules, the procedure of dispersing the collected particles in acetone and then collecting them was repeated 10times. Finally, the CuFB particles were dried under a vacuum overnight.

Supplementary Note 5 | Atomic structure of CuFB.

The H_2O-encapsulated structure of CuFB (str.**A**) was reported by Chen *et al*[2]. In this study, the CO_2-encapsulated structure of CuFB (str.**B**) was estimated based on str.**A**[2]. First, we performed peak indexing for the in situ XRD pattern of CuFB at 100 kPa CO_2 and 248 K using Conograph[3] software, and determined the cell parameters of str.**B** (Supplementary Table 3). Since these parameters are similar to those of str.**A**, the initial structure model of str.**B** was constructed by changing the cell parameters and replacing the H_2O molecules with the CO_2 molecules of str.**A**. Then, the geometry optimization for this model was performed using PreFerred Potential[4] version 4.0.0 in the Matlantis software (https://matlantis.com) integrated into the Atomic Simulation Environment[5] until the maximum force became smaller than 0.05 eV/Å. The simulated XRD pattern of the optimized structure model was in good agreement with the experimental patterns (Supplementary Fig. 13a). Thus, we considered this atomic model to be the CO_2-encapsulated structure of CuFB, without performing any further structural refinement, such as the Rietveld method.

The closed structure of CuFB was also reported by Chen *et al*[2]. Their analysis was performed at 110 K, and the cell parameters of their structural model (str.**C**) are listed in Supplementary Table 3. To investigate the closed structure at the measurement temperature for TRXRD (i.e., 248 K), peak indexing was performed on our XRD data and similar parameters to str.**C** were obtained. Then, by changing the cell parameters of str.**C** and performing geometry optimization using Matlantis, we obtained the closed structure model at 248 K (str.**C'**). The crystallographic information files for str.**B** and str.**C'** can be found in our data repository (https://github.com/2koza/framework-dependent-kinetics).

Supplementary Table 3 | Cell parameters for each structural model.

	str.**A**	str.**B**	str.**C**	str.**C'**
a [Å]	16.360	16.374	13.4864	13.440
b [Å]	10.753	11.092	11.1000	11.219
c [Å]	13.595	13.607	9.0925	9.253
α [°]	90	90	90	90
β [°]	105.302	99.103	120.335	121.095
γ [°]	90	90	90	90
space group	*C*2/*c*	*C*2/*c*	*C*2	*C*2

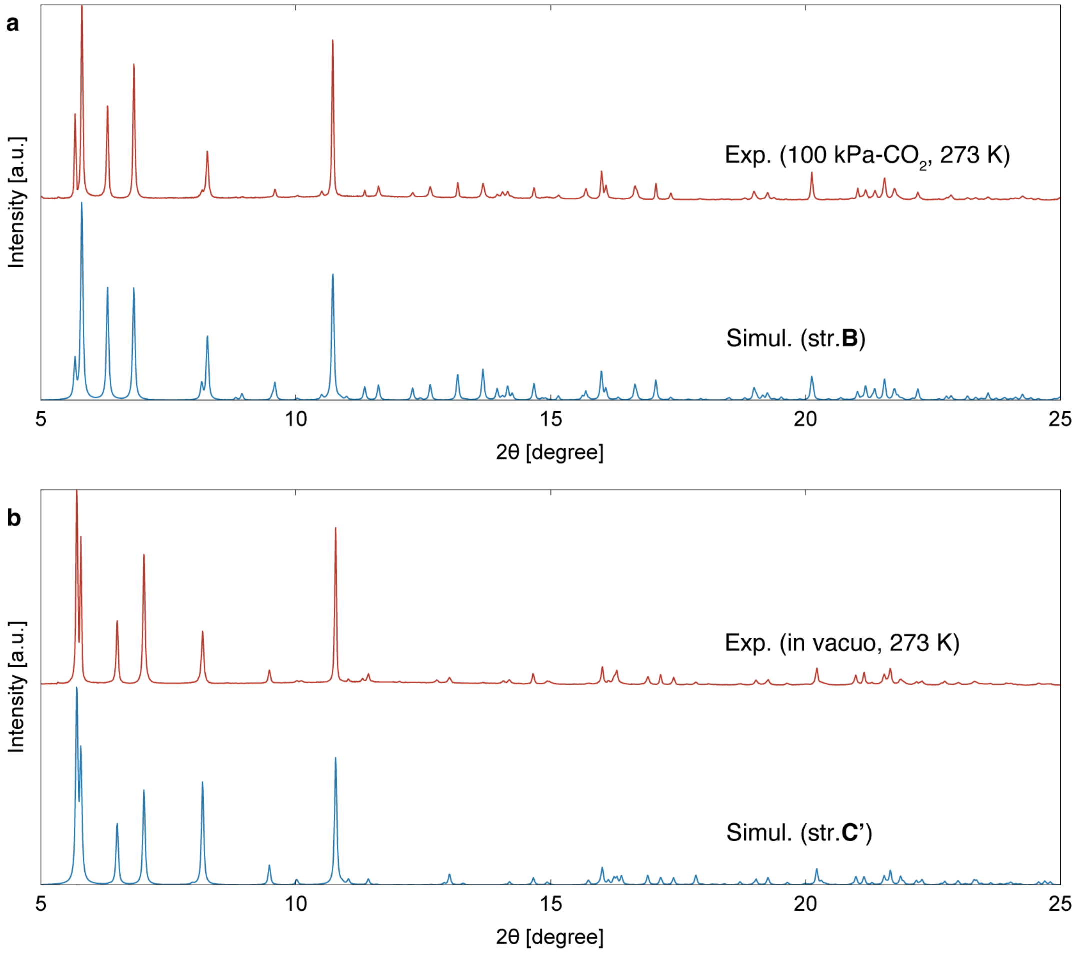

Supplementary Figure 13 | XRD patterns of CuFB. a, The experimental pattern at 100 kPa of CO_2 and 248 K and the simulated pattern of str.**B**. **b,** The experimental pattern at 248 K in vacuo and the simulated pattern of str.**C'**.

Reference

1 Kondo, A. *et al.* Gas Adsorption Mechanism and Kinetics of an Elastic Layer-Structured Metal–Organic Framework. *J. Phys. Chem. C* **116**, 4157–4162 (2012). https://doi.org:10.1021/jp210240x

2 Chen, B. *et al.* Rationally designed micropores within a metal-organic framework for selective sorption of gas molecules. *Inorg. Chem.* **46**, 1233–1236 (2007). https://doi.org:10.1021/ic0616434

3 Esmaeili, A., Kamiyama, T. & R., O.-T. New functions and graphical user interface attached to powder indexing software. *J. Appl. Crystallogr.* **50**, 651–659 (2017).

4 Takamoto, S. *et al.* Towards universal neural network potential for material discovery applicable to arbitrary combination of 45 elements. *Nat. Communs.* **13**, 2991 (2022). https://doi.org:10.1038/s41467-022-30687-9

5 Hjorth Larsen, A. *et al.* The atomic simulation environment—a Python library for working with atoms. *J. Phys.: Condens. Matter* **29**, 273002 (2017). https://doi.org:10.1088/1361-648X/aa680e
