# Supplementary material for: Generalised analytical method unravels framework-dependent kinetics of adsorption-induced structural transition in flexible metal–organic frameworks
Source: Nat Commun. 2023 Nov 8;14:6862. doi: 10.1038/s41467-023-42448-3 (PMC10632496; doi:10.1038/s41467-023-42448-3)
Supplement: Supplementary file 3 — Description of Additional Supplementary Files [file 41467_2023_42448_MOESM3_ESM.pdf]

Title: Supplementary Video 1.

Description: In situ optical microscopy movie of ELM-11 particles during the introduction of CO_2 at 0.8 kPa s^{-1} and ~297 K.

Title: Supplementary Video 2.

Description: The same video as Supplementary Video 1, but in each frame, the pixels that changed from the first frame (0 s) are coloured red.

Title: Supplementary Video 3.

Description: Schematic movement of the structural transition mechanism of CO_2 gate adsorption on ELM-11.

Title: Supplementary Video 4.

Description: In situ optical microscopy movie of MIL-53(Al) particles during the introduction of CO_2 at 0.8 kPa s^{-1} and ~297 K.

Title: Supplementary Video 5.

Description: The same video as Supplementary Video 4, but in each frame, the pixels that changed from the first frame (0 s) are coloured red.

Title: Supplementary Video 6.

Description: In situ optical microscopy movie of CuFB particles during the introduction of CO_2 at 0.8 kPa s^{-1} and ~297 K.

Title: Supplementary Video 7.

Description: The same video as Supplementary Video 6, but in each frame, the pixels that changed from the first frame (0 s) are coloured red.
